# Photon or Proton Therapy for Adolescent and Young Adult Tumors Focused on Long-Term Survivors

**DOI:** 10.7759/cureus.14627

**Published:** 2021-04-22

**Authors:** Masashi Mizumoto, Yoshiko Oshiro, Kayoko Tsujino, Shosei Shimizu, Takashi Iizumi, Haruko Numajiri, Kei Nakai, Toshiyuki Okumura, Toshinori Soejima, Hideyuki Sakurai

**Affiliations:** 1 Department of Radiation Oncology, University of Tsukuba Hospital, Tsukuba, JPN; 2 Department of Radiation Oncology, Tsukuba Medical Center Hospital, Tsukuba, JPN; 3 Department of Radiation Oncology, Hyogo Cancer Center, Akashi, JPN; 4 Department of Radiation Oncology, Proton Medical Research Center, University of Tsukuba Hospital, Tsukuba, JPN; 5 Department of Radiation Oncology, Kobe Proton Center, Kobe, JPN

**Keywords:** adolescent and young adult, radiotherapy, aya, proton beam therapy

## Abstract

Background

This study was conducted to evaluate late toxicities in adolescent and young adult (AYA) patients who received photon or proton therapy.

Methodology

A total of 106 AYA patients who received proton and photon therapy and were followed-up for more than two years were retrospectively evaluated. The median age of patients was 22 years (range, 15-29 years). A total of 47 patients were male and 59 were female. A total of 35 and 71 patients received photon and proton therapy, respectively. All but one patient received radiotherapy with curative intent. The target disease was benign and malignant in 28 and 78 patients, respectively.

Results

The median follow-up period in all patients was 62 months (range: 24-293 months). Grade 3 or higher toxicity was observed in 20 patients. There was one case of grade 5 toxicity (myelodysplastic syndrome), which was probably due to chemotherapy. No other secondary cancers were observed. Regarding life events, 15 and 88 patients were married and unmarried at the start of radiotherapy, respectively. Of the 88 unmarried patients, five were married after radiotherapy. Occupation and education were evaluated in 71 patients. Of the 71 patients, 33 were students, 21 were employed, and 16 were unemployed. Of the 33 students, eight were employed and 11 were at a higher educational grade after radiotherapy. Of the 21 employed patients, 17 had the same jobs and four had lost their jobs after radiotherapy. For the 16 unemployed patients, all remained unemployed.

Conclusions

This study is one of the largest studies to focus on life after radiation therapy among AYAs and suggests that cancer treatment has an influence on life events.

## Introduction

The definition of adolescent and young adults (AYAs) is different among countries [1]. In the United States, AYAs are patients aged between 15 and 39 years at cancer diagnosis, and in the United Kingdom, AYAs include patients aged between 13 and 24 years. However, a clear definition has not yet been established in other countries. For patients in these age groups, there is a gap in oncology services between pediatric and adult patients. Both rare and common malignancies are observed in AYAs. However, malignancies common among adults are not common in AYAs. Therefore, there are fewer studies providing information about malignancies in AYAs. For example, there is no clear consensus on whether AYAs with Hodgkin’s lymphoma should be treated with a pediatric or adult trial regimen; therefore, in 2018, the Children’s Oncology Group compared clinical trials and suggested that the treatment outcomes for AYAs are better in pediatric trials than adult trials [2].

In addition, late toxicities are a challenge for AYAs in association with improvement of treatment results. However, life stages rapidly change in the AYA age groups, and these patients face different psychosocial, emotional, and physical issues than pediatric and adult patients [3]. Therefore, we consider that adequate support is necessary for each personal situation.

In this study, we focused on life events, as well as late toxicities, in AYA patients who received photon and proton radiotherapy in two institutions in Japan.

## Materials and methods

A total of 106 AYA patients who received proton and photon therapy at two institutions in Japan were retrospectively analyzed. Photon and proton therapy were performed at separate institutes. Patients who received radiotherapy, were between 15 and 29 years of age, and were followed up for more than two years were enrolled in this study [4]. This study was approved by the institutional review board (Tsukuba Clinical Research & Development Organization; R01-144). Patient characteristics are shown in Table 1.

**Table 1:** Patient characteristics.

Characteristic		Number of patients
Sex	Male	47
	Female	59
Age (years)	Median	22
	Range	15–29
Disease	Benign	28
	Malignant	78
Radiotherapy	Photon	35
	Proton	71
Treatment intent	Curative	105
	Palliative	1

Overall, 35 and 71 patients received photon and proton therapy, respectively. Photon therapy was performed from 2004 to 2015 for 70 patients, and 35 of 70 patients were followed-up for less than two years (28 died due to cancer and seven were lost to follow-up). Proton therapy was performed from 1986 to 2017 for 100 patients. Of the 100 patients, 29 were followed-up for less than two years (17 died due to cancer and 12 were lost to follow-up). The median age of the patients was 22 years, ranging from 15 to 29 years. Of the 106 patients, 47 were male and 59 were female. All but one patient received radiotherapy with a curative intent. The only palliative treatment was photon radiotherapy with 34.4 Gy given in 23 fractions for central nervous system germinoma. The target disease was benign and malignant in 29 and 77 patients, respectively. Benign disease included arteriovenous malformation (n = 23), pituitary tumor (n = 3), meningioma (n = 1), schwannoma (n = 1), and thymoma (n = 1). Various malignant diseases were treated, especially with proton beam therapy (PBT). Photon radiotherapy mainly treated hematologic malignancies and cervical cancer. The details of the diseases are shown in Tables 2 and 3.

**Table 2:** Details of the diseases. ALL: acute lymphoblastic leukemia; AML: acute myelogenous (myeloid) leukemia; AVM: arteriovenous malformation; HCC: hepatocellular carcinoma; PNET: primitive neuroectodermal tumor

Disease	Total (n = 106)	Proton therapy (n = 71)	Photon therapy (n = 35)
ALL	4	0	4
AML	2	0	2
AVM	23	23	0
Brain tumor	20	14	6
Breast cancer	4	0	4
Cervical cancer	5	0	5
Chordoma	5	5	0
Chondrosarcoma	1	1	0
HCC	3	3	0
Hepatoblastoma	1	1	0
Head and neck cancer	5	2	3
Hodgkin’s lymphoma	5	3	2
Non-Hodgkin’s lymphoma	8	2	6
Neuroblastoma	2	2	0
Osteosarcoma	2	2	0
Rhabdomyosarcoma	4	4	0
PNET	3	1	2
Testicular tumor	2	1	1
Others	7	7	0

**Table 3:** Radiotherapy sites. TBI: total body irradiation: CSI: cerebrospinal irradiation

Radiotherapy site	Total (n = 106)	Proton therapy (n = 71)	Photon therapy (n = 35)
Brain	43	38	5
Skull base	6	6	0
Head and neck	12	7	5
Chest	18	10	8
Abdomen/pelvis	15	9	6
Extremities	1	1	0
Spine	1	0	1
TBI	9	0	9
CSI	1	0	1

The primary endpoint of this study was late toxicities, and the secondary endpoint was life events, including marriage, giving birth, and employment or education status. Toxicities were graded according to the Common Terminology Criteria for Adverse Events version 4.0 [5].

## Results

The median follow-up period in all patients was 62 months (range: 24.0-293.6 months) and 68 months for survivors. Late toxicities were observed in 45 patients. Details of the toxicities are shown in Table 4. Grade 3 or higher toxicity was observed in 20 patients. Grade 3 toxicity was seen in 17 patients, of which endocrine disorders occurred in eight patients who subsequently received hormonal replacement therapy. Other cases of grade 3 toxicity included gastrointestinal ulcer (n = 1), skin ulcer (n = 1), brain necrosis (n = 1), brainstem infarction (n = 1), low vision (n = 2), cataract (n = 2), and ileus (n = 1). Grade 4 toxicity was observed in two patients. A patient with moyamoya disease received total body irradiation at a dose of 12 Gy in four fractions for nasal natural killer (NK)/T-cell lymphoma when she was 29 years old. Facial bone deformity occurred in a patient who received photon therapy for nasal NK/T-cell lymphoma at a dose of 50 Gy in 25 fractions when he was 22 years old. Grade 5 toxicity in the form of myelodysplastic syndrome occurred in a patient who received proton therapy for Ewing sarcoma (local irradiation of 55.8 Gy (RBE) in 31 fractions) when she was 15 years old. Secondary cancer was not observed outside of the one case of myelodysplastic syndrome. Depression was observed in two patients.

**Table 4:** Toxicities observed in patients. CVD: cardiovascular disease

Grade	1	2	3	4	5
Skin atrophy/ulcer	1	3	1	0	0
Facial deformation	0	0	0	1	0
Cataract/low vision	0	0	4	0	0
Ear (noise, hearing disorder, otitis)	1	3	0	0	0
Dry mouth	2	0	0	0	0
Hoarse voice	1	0	0	0	0
Seizures	0	5	0	0	0
Headache	1	3	0	0	0
Brain atrophy	1	0	0	0	0
Brain necrosis	0	0	1	0	0
CVD (moyamoya disease, infarction)	0	0	1	1	0
Endocrine disorder	0	0	8	0	0
Pneumonia	1	0	0	0	0
Enteritis/gastrointestinal perforation	0	1	1	0	0
Ileus	0	0	1	0	0
Extremity pain	0	1	0	0	0
Depression	0	2	0	0	0
Lymphedema	2	0	0	0	0
Myelodysplastic syndrome	0	0	0	0	1
Total	10	18	17	2	1

Regarding life events, 15 and 88 patients were married and unmarried at the start of radiotherapy, respectively, and three had unknown marital status. Of the 88 unmarried patients, five were married after radiotherapy, and 83 were unmarried at the final follow-up.

A total of 90 patients did not have a child at the start of radiotherapy. Of the 90 patients, 45 were female, and five gave birth after radiotherapy. No male patients had children at the start of the treatment.

**Figure 1:**
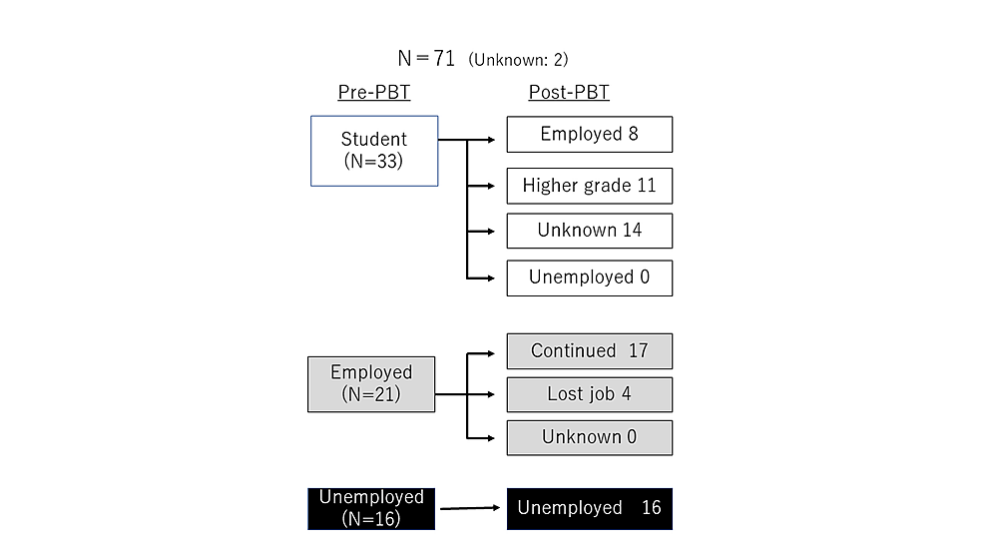
Employment and education status of 71 patients.

Occupation and education were evaluated in 71 patients who received PBT. Of the 71 patients, 33 were students, 21 were employed, and 16 were unemployed at the start of radiotherapy. Occupational information was unknown for the remaining two patients. Of the 33 student patients, eight (24%) were employed, 11 (33%) were at a higher grade of education at the last follow-up, and 14 had unknown educational status. Of the 21 employed patients, 17 (81%) were still in the same jobs at the last follow-up, but four had lost their jobs. Of the 16 unemployed patients, all were still unemployed at the final follow-up (Figure 1).

## Discussion

In Japan, cancer morbidity is increasing in those over 45 years old [6] and cancer mortality rate is increasing in those over 55 years old [7]. Hence, AYAs are rare among patients with common cancers. Furthermore, life stages rapidly change in the AYA generation and differ from those in other generations. Adolescents are in a stage of mental and social development and have not started working yet or have not become economically independent. Young adults start to become independent and start working. Therefore, the treatment effects of the same radiotherapy regimen may differ based on their circumstances. Naturally, AYA cancer survivors experience cancer-related distress [8-13], such as fear of cancer recurrence. Additionally, many factors influencing this distress have been suggested, including multimodal treatment, history of psychological distress [10], employment, type of cancer [9], age [11], and gender [8]. Wang et al. evaluated cancer-related worry in 15-26-year olds using the six-item Cancer Worry Scale [8]. They concluded that female survivors and higher treatment intensity were associated with increased cancer worry regarding infertility and/or secondary cancer. Mattoson et al. also reported that 85% of the patients, suffering from gynecological cancer at 19-39 years, were suffering from cancer-related distress, including fear of cancer recurrence (61%), anxiety (53%), depression (51%), fear of death (32%), concerns regarding sexuality (34%), fertility (27%), and change in body image (27%) [10]. Shay et al. showed that the rate of fear of recurrence was significantly higher among AYA survivors than among those diagnosed at an older age [9]. Beattie et al. also showed that younger age was associated with higher fear of recurrence among breast cancer patients [11]. In our hospital, we performed follow-up for pediatric patients with a radiation oncologist, pediatrician, and/or surgeon to respond to various changes. Similar to dealing with pediatric patients, follow-up by multiple experts (radiation oncologist, clinical psychologist, social worker, gynecologist, etc.) may be needed for AYA cancer survivors to reduce cancer-related events.

The Children’s Oncology Group recommends that patients receiving radiotherapy to the head and neck undergo dental examination and cleaning every six months because of the risk of dental decay [14]. Hamilton et al. reported late effects in AYA head and neck cancer survivors who survived for more than five years after diagnosis. In this study, 78% of the survivors had at least one late toxicity from radiotherapy, including chronic skin changes, osteonecrosis, and hearing loss. Additionally, a second malignancy was observed in 17% of the patients. Lim et al. reported the results of treatment of 176 AYAs with brain and skull base tumors at the Paul Scherrer Institute [15]. PBT is expected to have a lower rate of toxicities due to its favorable dose distribution, and severe (greater than grade 3) ototoxicity and neurotoxicity were observed in 3.4% and 2.9% of the patients, respectively. However, the rate of unemployment was 9.5% pre-PBT, increasing up to 23.8% post-PBT. Adverse events caused by PBT and having to take time for surgery and PBT affected employment.

In this study, 76% of the patients who were students could find jobs or continued higher-grade education, and 81% of the patients who had a job continued their previous jobs. However, none of the patients who were unemployed at the time of radiotherapy could find a job after radiotherapy. This result may be due to the lifetime employment system in Japan, and suggests that once a patient loses their job, it is difficult for them to find new jobs. In addition, the ratio of number of marriages to women giving birth was also low. Regarding birth or marriage, Gerstl et al. conducted a meta-analysis of reproduction among AYA female cancer survivors. They suggested a low rate of live births for patients who received chemotherapy or radiotherapy compared to surgery alone: 10% among patients who received chemotherapy alone, 18% among patients who received radiotherapy alone, and 44% among patients who received surgery alone. They also suggested that among AYA patients who became pregnant, 79% gave birth, and 22% of these births were preterm [16]. In our study, marriage and birth rates were low. At the last follow-up, 83 of the 88 patients were unmarried and five of the 45 female patients gave birth after radiotherapy. Some studies have shown that PBT probably reduces the risk of late toxicity among younger patients [17-19]. However, we did not compare patients who received PBT and photon radiotherapy because the disease and tumor location were very different among patients treated with these two modalities, and no secondary malignancies were observed in this study, possibly due to the short follow-up period.

The main limitations of this study are its retrospective design and the short follow-up period.

## Conclusions

This is one of the largest studies focusing on life after radiation therapy, suggesting that cancer treatment may impact life events. More detailed data collected over a longer period of time are necessary to clarify the effect of radiotherapy on the future lives for AYAs.
